# A role for the mitochondrial-associated protein p32 in regulation of trophoblast proliferation

**DOI:** 10.1093/molehr/gau039

**Published:** 2014-05-29

**Authors:** P. Matos, J.A. Horn, F. Beards, S. Lui, M. Desforges, L.K. Harris

**Affiliations:** 1 Maternal and Fetal Health Research Centre, Institute of Human Development, University of Manchester, Manchester M13 9WL, UK; 2 Maternal and Fetal Health Research Centre, St. Mary's Hospital, Central Manchester University Hospitals NHS Foundation Trust, Manchester Academic Health Science Centre, Manchester M13 9WL, UK

**Keywords:** fetal growth restriction, mitochondria, p32, proliferation, trophoblast

## Abstract

p32 is a conserved eukaryotic protein which is primarily expressed in the mitochondria and regulates cell proliferation, migration and metabolism in various tissues. In this study, we sought to examine the expression and function of p32 in the human placenta. p32 was highly expressed in the syncytiotrophoblast, the underlying cytotrophoblast (CTB), the vascular endothelium and by a proportion of cells in the villous stroma in first trimester and term placenta. p32 mRNA and protein expression was significantly higher in the first trimester of pregnancy than at term, and expression in the trophoblast was significantly reduced in placentas from women with fetal growth restriction (FGR). Small interfering RNA (siRNA)-mediated knockdown of p32 in term placental explants significantly reduced the number of Ki67-positive CTB, but did not alter CTB apoptosis or necrosis. p32 knockdown increased lactate production, reduced glucose extraction from culture medium and was associated with reduced MitoTracker dye accumulation in trophoblast mitochondria. p32 knockdown was also associated with a significant reduction in expression of the mitochondrial respiratory complexes I and IV. These data suggest that p32 expression is important for CTB proliferation, via a mechanism involving regulation of normal mitochondrial function. As p32 expression is reduced in FGR placentas, this may contribute to some of the observed placental pathology, such as reduced CTB proliferation and mitochondrial dysfunction.

## Introduction

p32, also known as p33, hyaluronic acid binding protein 1 (HABP1), receptor for the globular head domains of complement C1q (gC1qR) and C1q binding protein (C1QBP), is a highly conserved 32 kDa eukaryotic protein. It is primarily localized to the mitochondrial matrix (Muta *et al.*, 1997), but has been identified in other subcellular compartments, including the nucleus, endoplasmic reticulum, Golgi and on the cell surface (Dedio *et al.*, 1998; Ghosh *et al.*, 2004; Sengupta *et al.*, 2005; Fogal *et al.*, 2008; Hu *et al.*, 2013). p32 exists as a highly acidic, doughnut-shaped trimer that interacts with hyaluronic acid, the HIV Tat-associated protein, complement C1q and the tumour suppressor ARF, suggesting a role as a multifunctional chaperone protein (Yu *et al.*, 1995; Ghebrehiwet and Peerschke, 2004; Peerschke and Ghebrehiwet, 2007). Other studies have implicated p32 as a key regulator of cell proliferation, adhesion, migration and invasion, especially in metabolically active and rapidly growing tissues (Feng *et al.*, 2002; Kim *et al.*, 2011). p32 expression is elevated tumours of the breast, epidermis and ovary (Fogal *et al.*, 2008; Chen *et al.*, 2009; Kim *et al.*, 2011; Yu *et al.*, 2013; Zhang *et al.*, 2013), and increased expression correlates with malignancy (Chen *et al.*, 2009; Yu *et al.*, 2013). Association of p32 with the cell membrane has been shown to promote migration and invasion *in vitro* (Prakash *et al.*, 2011) and may aid tumour cells in evading the complement system by inhibiting the haemolytic activity of C1q (Rozanov *et al.*, 2002). p32 is also functionally important for the maintenance of oxidative phosphorylation (Muta *et al.*, 1997); stable knockdown of p32 expression in tumour cell lines leads to a reduction in proliferation, impairment of mitochondrial function and shifts metabolism from oxidative phosphorylation to glycolysis (Fogal *et al.*, 2010).

The placenta is highly metabolically active organ, which undergoes rapid growth during its 9-month lifespan. Within the placenta, a progenitor cell population of villous cytotrophoblasts (CTBs) proliferates rapidly to facilitate expansion of the overlying, post-mitotic syncytiotrophoblast (STB) (Mayhew, 2001). The STB is a specialized, multinucleate, solute-transporting epithelium with endocrine/paracrine functions that facilitates delivery of nutrients to the fetus and produces hormones that sustain pregnancy (Desforges and Sibley, 2009). The STB is renewed throughout pregnancy by cellular turnover, a process involving CTB proliferation followed by differentiation and subsequent fusion with STB. During the first trimester of pregnancy, villous CTB also give rise to a population of migratory extravillous trophoblast (EVT), which invade the maternal decidua and colonize the uterine spiral arterioles (Pijnenborg *et al.*, 2006).

The processes of trophoblast proliferation, differentiation and invasion must be tightly regulated so that the villous architecture is maintained whilst placental growth keeps pace with the needs of the developing fetus. Dysregulation of trophoblast turnover is associated with pregnancy complications including fetal growth restriction (FGR; Macara *et al.*, 1996; Chen *et al.*, 2002; Levy *et al.*, 2002; Heazell *et al.*, 2010). The placenta is also a highly metabolically active organ; efficient mitochondrial function is crucial to provide support for energetically demanding processes such as CTB proliferation, nutrient transport and protein synthesis (Murray, 2012). As p32 regulates proliferation and metabolism in a number of cell types, we hypothesized that p32 expression would be important for normal trophoblast turnover and mitochondrial function. Here, we demonstrate that p32 is highly expressed in the trophoblast of human placenta and that siRNA-mediated knockdown of p32 leads to a reduction in CTB proliferation and impaired mitochondrial function.

## Materials and Methods

### Materials

Unless stated otherwise, all materials used were obtained from Sigma-Aldrich (Poole, UK).

### Human tissue collection

Human placentas were obtained from elective medical or surgical termination of pregnancy during the first trimester (5–12 weeks) or second trimester (13–19 weeks). Placentas from uncomplicated pregnancies (33.0, 37.4, 37.9, 38.6, 39.0, 39.0, 39.3, 39.3, 39.3, 39.4, 39.7 and 41.7 weeks gestation) and placentas from women with FGR (below fifth birthweight centile; 31.7, 36.6, 37.9, 38.3, 38.6, 38.6, 39.1, 39.1, 39.7 and 41.9 weeks gestation) were collected within 30 min of vaginal or elective Caesarean delivery. Written informed consent was obtained and the study had local research ethics committee approval (08/H1010/28; 08/H1010/55; 08/H1011/83).

### Villous explant culture

Villous tissue from normal term placentas was randomly sampled and washed several times under sterile conditions, in a 1:1 ratio of serum-free Dulbecco's modified Eagle medium (DMEM) and Ham's F12 containing penicillin (100 IU/ml), streptomycin (100 ug/ml) and amphotericin B (2.5 ug/ml) (Lonza Biosciences, UK). Explants of ∼2 mm^3^ were dissected and placed into 24-well culture plates (1/well), precoated with 1% (w/v) agarose. Explants were submerged in 1 ml of DMEM/Ham's F12 containing glutamine (2 mM), penicillin (100 IU/ml), streptomycin (100 ug/ml) and 10% (v/v) fetal bovine serum (Invitrogen, UK). The tissue was maintained in 95% air and 5% CO_2_ at 37°C for up to 72 h.

### Transfection of villous explants

Knockdown of p32 in the STB layer of term placental explants was attained using two different small interfering RNA (siRNA) sequences (Stealth Select RNAi™ siRNA; sequence 2: #HSS186347; sequence 3: #HSS101146, Invitrogen, UK; denoted in the manuscript as siRNA 1 and siRNA 2, respectively). A non-targeting (NT) siRNA was used as a negative control (#12935300; Invitrogen). siRNA (50 nM) was added to the culture medium in the absence of any transfection reagents and tissue was cultured for up to 72 h, as previously described (Forbes *et al.*, 2009; Harris *et al.*, 2010). The wet weight of each explant was noted and the culture media was collected, centrifuged (13 225*g*, 5 min) to remove any debris and stored at −20°C. Explants were either submerged in RNALater (Sigma-Aldrich) for 24 h, then stored at −80°C, or were fixed in neutral buffered formalin [4% (v/v); 24 h; Sigma-Aldrich], subjected to routine histological processing and embedded in paraffin wax.

### Real-time quantitative PCR

Total RNA was extracted from first trimester and term placentas, or term placental explants using a MirVana™ miRNA Isolation Kit (which isolates mRNA and miRNA simultaneously; Life Technologies Ltd, UK), according to the manufacturer's instructions. Total RNA was quantified using Quant-iT™ Ribogreen RNA Assay Kit (Invitrogen). Total RNA (50 ng) was reverse transcribed using an AffinityScript Multiple Temperature cDNA Synthesis kit (Agilent; UK). Quantification of p32 cDNA was performed using Brilliant III Ultra-Fast SYBR Green QPCR Master Mix (Agilent) with primers obtained from Invitrogen (p32 forward: 5′-TCAACTCCCAATTTCGTGGTT-3′, p32 reverse: 5′-TCCTCTGGATAATGACAGTCCAA-3′) and using 5-carboxy-x-rhodamine (ROX) as a passive reference dye in a Mx3000p or a Mx3005p QPCR machine (Stratagene). QPCRs were performed in triplicate and a set of human reference RNA standards (Agilent) was included in every reaction. These were used to construct a standard curve, from which the concentration of p32 mRNA was quantified. In parallel, qPCR for the housekeeping genes 18S ribosomal RNA (forward: 5′-GCTGGAATTACCGCGGCT-3′, reverse: 5′-CGGCTACCACATCCAAGGAA-3′) and YWHAZ (forward: 5′-ACTTTTGGTACATTGTGGCTTCAA-3′, reverse: 5′-CCGCCAGGACAAACCAGTAT-3′) was performed on the same samples and standards, to quantify their expression. p32 expression was then normalized to 18S or YWHAZ expression (Harris *et al.*, 2010; Desforges *et al.*, 2013).

### Immunohistochemistry

Sections (5 µm) of wax-embedded placenta were dewaxed and rehydrated using Histoclear and decreasing concentrations of ethanol, after which they were submerged in distilled water for 5 min, then microwaved at full power for 10 min in 0.01 M sodium citrate buffer, pH 6.0. After cooling, the sections were washed in distilled water and endogenous peroxidase activity was quenched by incubation with 3% (v/v) hydrogen peroxide at room temperature for 10 min. Sections were washed in Tris-buffered saline (TBS, 2 × 5 min) then incubated with 5% (w/v) bovine serum albumin in TBS for 30 min at room temperature. Primary antibodies were diluted in TBS as follows: rabbit anti-human p32 (0.15 µg/ml, #HPA026483, Sigma-Aldrich); mouse anti-human Ki67 (35 µg/ml, clone MIB-1, #M7240, Dako); mouse anti-human cleaved cytokeratin-18 (M30 CytoDeath kit, #12-140-322-001; 1:100, Roche) and applied to individual sections. Isotype control mouse and rabbit IgG (Sigma-Aldrich, #I8765 and #I8140) were diluted to the same working concentration. Slides were incubated overnight at 4°C in a humidity chamber. Slides were washed in TBS (3 × 5 min), biotinylated anti-rabbit and anti-mouse secondary antibodies (#E0431 and #E0433; Dako UK) were diluted in TBS to 3.85 and 4.4 µg/ml, respectively, then were applied to the sections and incubated for 30 min at room temperature. Slides were washed again with TBS as described above, followed by incubation with avidin peroxidase (5 µg/ml) for 30 min at room temperature. Another series of washes followed, and sections were incubated with the chromogen diaminobenzidine (DAB 0.05% (w/v); H_2_O_2_ 0.015% (v/v)). All tissue sections were incubated with DAB for the same length of time so that comparisons could be made between individual samples, and all slides were stained in a single run to eliminate inter-experimental variations in staining intensity. Sections were then washed with distilled H_2_O, counterstained with Harris' haematoxylin and washed briefly in acid/alcohol solution. Finally, sections were washed in warm tap H_2_O, dehydrated in increasing concentrations of ethanol and Histoclear and mounted with DPX for imaging using Leitz Dialux22 and Olympus Bx41 light microscopes. Exposure times and background colour were matched at image capture. Semi-quantitative analysis of syncytial p32 staining intensity was performed by two independent, blinded observers: three random images of each immunostained sample were captured, images were assigned a score between 0 (no staining) and 3 (intense staining), and a mean value was calculated for each sample. To quantify CTB proliferation and apoptosis following p32 knockdown, six random images of each immunostained explant were captured. The number of Ki67-positive or M30-positive CTB was counted and expressed as a percentage of the total number of CTB in the field of view; these data were used to determine a mean number of positive CTB for each explant (Harris *et al.*, 2010).

### Western blotting

Villous tissue from first trimester, term or FGR placentas, or term placental explants transfected with siRNA, were washed in phosphate-buffered saline (PBS). Tissue was homogenized in lysis buffer (1 ml/sample) containing protease and phosphatase inhibitors [1 × PBS, 1% (v/v) NP-40, 0.5% (w/v) sodium deoxycholate, 0.1% (w/v) SDS, 1 mM Na_3_VO_4_, 1 mM PMSF, 10 µg/ml aprotinin] and the resulting lysates were incubated on ice for 20 min, then centrifuged at 13 000*g*, 10 min. The resulting supernatants were removed and stored at −20°C. Equal amounts of protein (20 μg) were separated by SDS–PAGE on 10% gels and transferred to polyvinylidene difluoride membranes. Membranes were blocked [TBS, 0.1% (v/v) Tween, 5% (w/v) milk powder] for 1 h, and then probed overnight with a rabbit anti-human p32 (1:1000; #HPA026483, Sigma-Aldrich), a mouse anti-human MitoProfile Total OXPHOS antibody cocktail (1:500; ab110411, Abcam), a mouse anti-human VDAC antibody (1:500; #4866, Cell Signalling), a rabbit anti-human GAPDH antibody (1:500; #G9545, Sigma-Aldrich) or a mouse anti-human β-actin antibody (1:1000; #A5441, Sigma-Aldrich). After washing in TBS Tween [TBS containing 0.1% (v/v) Tween; 3 × 10 min], membranes were incubated with an anti-rabbit or anti-mouse HRP-conjugated secondary antibody (1:1250; 1 h; #P0447 and #P0448, Dako). After further washes in TBS Tween, proteins were detected by enhanced chemiluminescence (GE Healthcare, Chalfont St. Giles, UK). Blots were scanned and immunoreactive bands analysed for mean signal intensity using Image J software. p32 expression in first trimester and term tissue was normalized to β-actin, as expression of this housekeeping gene was not altered by gestational age (Supplementary data, Fig. S1). p32 expression in healthy term and FGR samples was normalized to the housekeeping gene glyceraldehyde 3-phosphate dehydrogenase (GAPDH), as expression of this housekeeping gene was not altered by placental pathology; during the course of this study we observed that β-actin protein expression was significantly reduced in our FGR tissue lysates, compared with normal term placental lysates (data not shown). OXPHOS proteins were normalized to the ubiquitously expressed mitochondrial porin protein VDAC.

### Lactate dehydrogenase assay

Lactate dehydrogenase (LDH) in explant culture medium was measured using Cytotoxicity Detection KitPlus (LDH; Roche, UK) following the manufacturer's instructions. Samples were thawed on ice and were transferred in triplicate (100 µl/well) to a 96-well plate. Reaction mix (100 µl/well) was added, samples were incubated for 15 min at room temperature, then 50 µl of stop solution was applied. Absorbance at 490 nm was quantified using a plate reader (Versamax, Molecular Devices, Wokingham), and data were normalized to the wet weight of the explants.

### Glucose assay

Glucose concentration in explant culture medium was quantified as previously described (Hulme *et al.*, 2012). A buffer/chromophore reagent was prepared by mixing an equal volume (3.5 ml) of 4-aminoantipyrine (10 mM) and *N*-ethyl-*N*-sulfopropyl-*m*-toluidine (10 mM) with 3.0 ml of 0.8 M sodium phosphate buffer (0.8 M; pH 6.0). 100 μl of reagent was added to 10 μl horseradish peroxidase (1.6 units/ml) and 2 μl of sample or standard (0–50 mM d-glucose) in a 96-well plate. The reaction was initiated by the addition of 10 μl glucose oxidase (2.7 units/ml) and the plate was incubated at room temperature for 10 min. Absorbance at 550 nm was quantified using a plate reader. Concentration was calculated using a standard curve constructed using defined concentrations of d-glucose, and data were normalized to the wet weight of the explants.

### Lactate assay

Lactate concentration in explant culture medium was quantified using a commercially available fluorimetric lactate assay kit (Abcam). Samples were transferred in triplicate (50 µl/well) to a 96-well plate, reaction mix (50 µl/well) was added and samples were incubated for 30 min in the dark. Fluorescence at 590 nm was measured using a plate reader (Versamax), and data were normalized to the wet weight of the explants.

### MitoTracker uptake assay in term placental explants

Term placental explants were transfected with NT or p32-specific siRNA sequences and cultured for 72 h, then were subjected to a pulse-chase experiment. Explants were incubated with MitoTracker Red CMXRos (10 nM; Life Technologies) for 6 h to allow passive transfer into the syncytium, then were transferred to fresh culture medium for a further 18 h to allow accumulation of dye in the mitochondria to occur (i.e. up to 96 h post-transfection). MitoTracker Red CMXRos is a mitochondrial membrane potential-sensitive, red fluorochrome that accumulates in active mitochondria (Pendergrass *et al.*, 2004). Explants were washed in PBS, fixed in neutral buffered formalin [4% (v/v); 24 h] and embedded in Optimal Cutting Temperature cryopreservation medium (RA Lamb; UK). Tissue sections (10 µm) were fixed in ice-cold methanol, washed twice in PBS (2 × 5 min), mounted with Vectashield mounting medium containing 4′,6-diamidino-2-phenylindole (DAPI; Vector Laboratories, UK) and visualized using a Zeiss Axiovision fluorescence microscope. Images were captured at the same exposure so that comparisons across samples could be made.

### Data analysis

Data were analysed using GraphPad Prism software (Version 5; GraphPad, CA). Non-parametric data were expressed as medians and analysed by Mann–Whitney *U*-test (unpaired data), Wilcoxon matched-pairs test (paired data) or a Kruskal–Wallis test. Data from a minimum of three independent experiments is presented. Significance was taken as *P* < 0.05.

## Results

### p32 expression in human placenta

**Figure 1:**
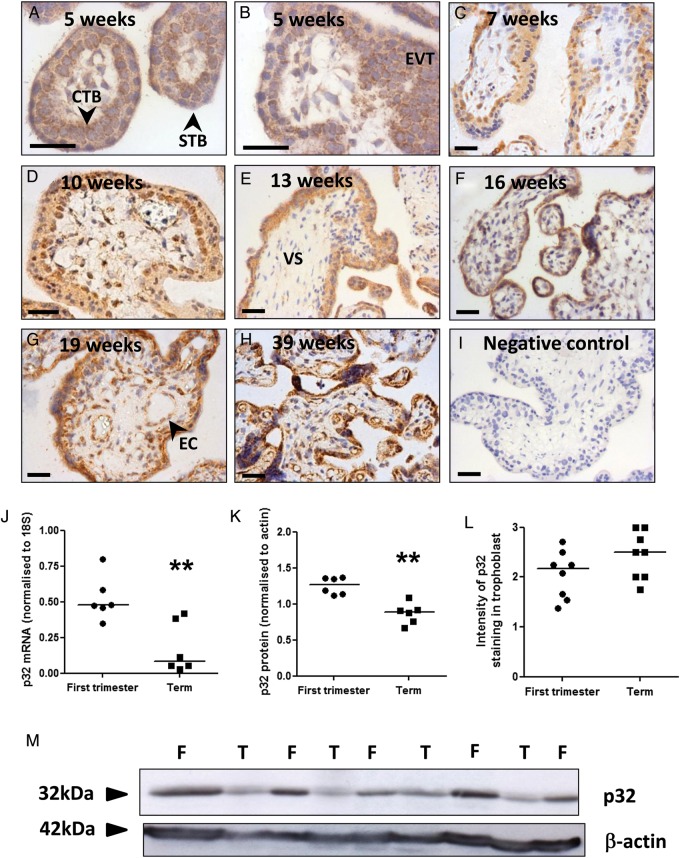
p32 expression in human placenta. Immunohistochemical analysis of (**A**–**D**) first trimester, (**E**–**G**) second trimester and (**H**) term placenta stained with an antibody to p32 or (**I**) isotype control IgG (DAB labelling, brown). Nuclei were counterstained with haematoxylin (blue). Scale bar represents 50 µm. Images are representative of *n* = 12 samples. STB, syncytiotrophoblast; CTB, cytotrophoblast; EVT, extravillous trophoblast; VS, villous stroma; EC, endothelial cells. (**J**) p32 mRNA expression in first trimester and term placenta was assessed by quantitative PCR and normalized to expression of 18S ribosomal RNA (median, *n* = 6; **P < 0.01, Mann–Whitney *U*-test). (**K**) p32 protein expression in first trimester (F) and term (T) placenta was analysed by western blotting, quantified by densitometry and normalized to β-actin expression (median, *n* = 6; **P < 0.01, Mann–Whitney *U*-test). (**L**) Trophoblast p32 staining intensity was quantified by two independent assessors. Median, *n* = 8. (**M**) p32 western blot.

In first trimester placenta, punctate expression of p32 was observed in villous CTB, throughout the STB layer and in EVT within cell columns (Fig. 1A–D; Supplementary data, Fig. S2A–D). The vascular endothelium of placental vessels and a subset of cells within the villous stroma were also immunopositive. p32 expression was also observed in the STB, CTB, vascular endothelium and villous stroma of second trimester and term placentas (Fig. 1E–H; Supplementary data, Fig. S2E–H). Quantitative RT–PCR and western blotting demonstrated that total p32 mRNA and protein levels were significantly reduced at term, compared with first trimester (Fig. 1J, K and M, Supplementary data, Fig. S3; **P < 0.01). However, p32 expression in the trophoblast bilayer remained high-throughput gestation, and semi-quantitative analysis of the immunostaining showed that p32 expression in the trophoblast was not affected by gestational age (Fig. 1L).

### Trophoblast p32 expression is reduced in placentas from women with FGR

**Figure 2:**
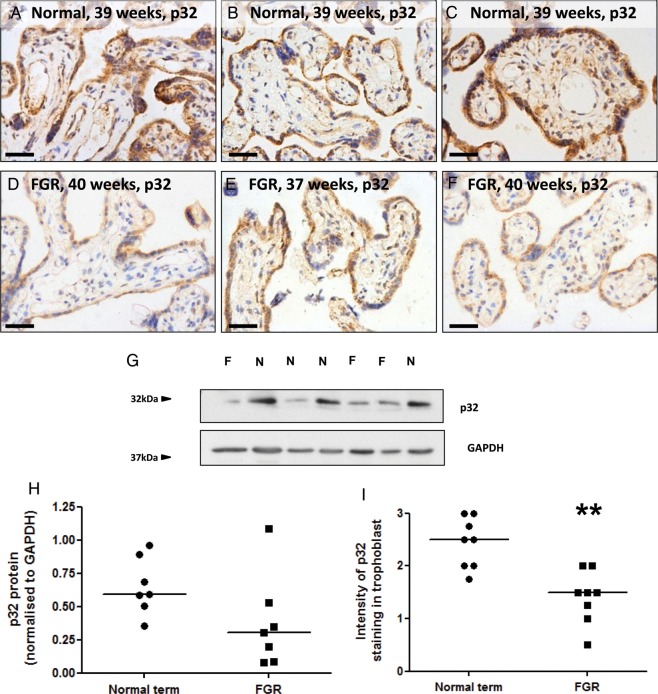
Trophoblast p32 expression is reduced in placentas from women with FGR. (**A**–**F**) Immunostaining of (A–C) normal term placentas or (D–F) placentas from women with FGR with an antibody to p32 (DAB labelling, brown). Nuclei were counterstained with haematoxylin (blue). Scale bar represents 50 µm. Images are representative of *n* = 8 normal and eight FGR samples. (**G**) Total p32 expression in normal term placentas (*n* = 7) or placentas from women with FGR (*n* = 7) was analysed by western blotting. (**H**) Expression was quantified by densitometry and normalized to GAPDH expression. Median, *n* = 7. (**I**) Trophoblast p32 staining intensity was quantified by two independent assessors. Median, *n* = 8; **P < 0.01, Mann–Whitney *U*-test.

p32 protein expression was compared in matched placental samples from healthy pregnant women and women with FGR. Patient demographics are shown in Table I. The STB, CTB, vascular endothelium and some cells in the villous stroma where immunopositive; however, p32 expression in the trophoblast bilayer was reduced in placentas from women with FGR (Fig. 2A–F). Total p32 expression, as measured by western blotting, varied between individual placentas in both patient groups but was not significantly reduced in FGR lysates (Fig. 2G and H). However, semi-quantitative assessment of p32 immunostaining by two independent observers concluded that p32 expression in the trophoblast was significantly reduced in the placentas of women with FGR (Fig. 2I).

**Table I:** Patient demographics.

	Normal (*n* = 12)	FGR (*n* = 10)
Age (years)	33.5 (25–41)	29.5 (22–35)
BMI (kg/m^2^)	23.7 (17.4–33.7)	23.9 (22.6–37)
Birthweight (g)	3420 (2100–4020)	2360 (942–3100)***
Birthweight centile	46.5 (37.9–99.8)	0.83 (0–3)***
Gestation at delivery (weeks)	39.1 (33–41.7)	38.6 (31.7–41.9)
Caesarean section	6 (50%)	8 (80%)
Male fetus	5 (42%)	4 (40%)
Smoker	3 (25%)	3 (30%)

Continuous data are presented as median and range.

***P < 0.001, Mann–Whitney *U*-test.

### siRNA-mediated knockdown of p32 in term placental explants

**Figure 3:**
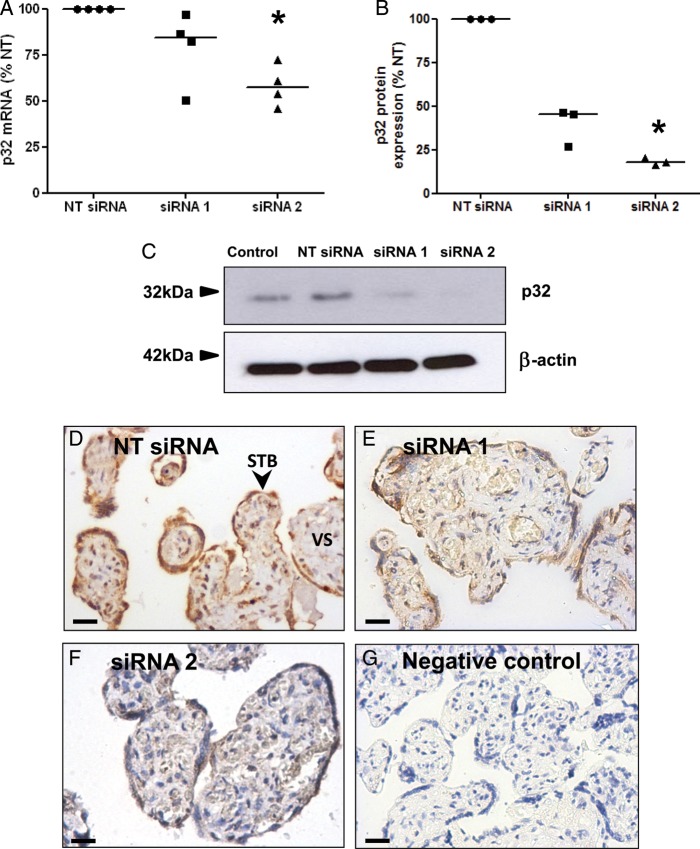
siRNA-mediated knockdown of p32 in term placental explants. Term placental explants were transfected with a non-targeting siRNA sequence (NT; 50 nM) or two different siRNA sequences specific for p32 (siRNA 1, siRNA 2; 50 nM). After 72 h, p32 expression was assessed by (**A**) quantitative PCR (median, *n* = 4) *P < 0.05, Kruskal–Wallis test (**B** and **C**), western blotting (median, *n* = 3; β-actin was used as a loading control), *P < 0.05, Kruskal–Wallis test and (**D**–**G**) immunostaining using a p32-specific primary antibody or control IgG (DAB labelling, brown; *n* = 4). Nuclei were counterstained with haematoxylin (blue). Scale bar represents 50 µm. STB, syncytiotrophoblast; VS, villous stroma.

To recapitulate the reduced syncytial expression of p32 observed in FGR placentas and to determine whether p32 regulates CTB function, p32 protein expression was knocked down in term placental explants using two p32-specific siRNA sequences (siRNA1, siRNA2). A non-targeting (NT) siRNA sequence served as a control. Transfection was performed in the absence of transfection reagents, as this method results in efficient knockdown of syncytial proteins (Forbes *et al.*, 2009; Harris *et al.*, 2010). Both p32-specific sequences reduced target mRNA (siRNA 1: 21.0% reduction versus NT; siRNA2: 41.8% reduction; Fig. 3A) and protein expression (siRNA 1: 61% reduction versus NT; siRNA2: 82.0% reduction; Fig. 3B and C). Immunohistochemical analysis of explants transfected with p32-specific siRNA demonstrated that trophoblast p32 expression was greatly reduced (Fig. 3D–G). Knockdown was also evident in some areas of the villous stroma, and was more frequently observed in explants treated with siRNA2; however, stromal p32 expression was highly variable.

### p32 knockdown reduced CTB proliferation in term placental explants

**Figure 4:**
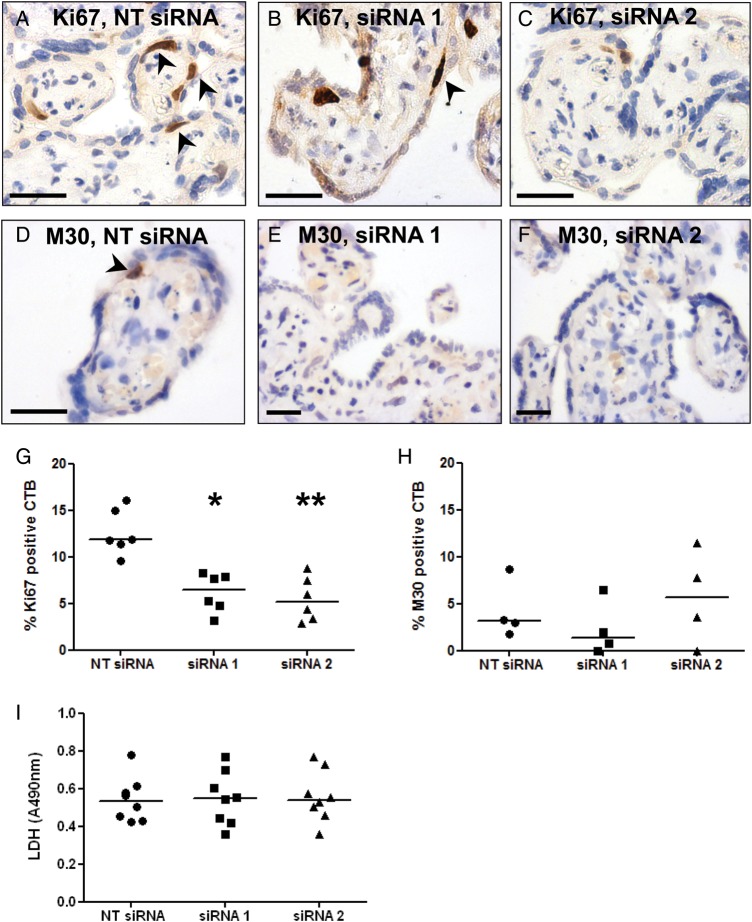
p32 knockdown alters CTB proliferation. Term placental explants were transfected with a non-targeting siRNA sequence (NT; 50 nM) or two different siRNA sequences specific for p32 (siRNA 1, siRNA 2; 50 nM). After 72 h, proliferation and apoptosis were assessed by immunohistochemical analysis of (**A**–**C**) Ki67- and (**D**–**F**) M30-positive CTB. Arrowheads denote positive cells. (**G**) Percentage of Ki67-positive CTB. Median, *n* = 6; *P < 0.05, **P < 0.01, Kruskal–Wallis test. (**H**) Percentage of M30-positive CTB. Median, *n* = 4. (**I**) Necrosis was assessed by measuring LDH release into the culture medium. Median, *n* = 8.

Following p32 knockdown, explants were immunostained with antibodies to Ki67 and M30, to assess rates of CTB proliferation and apoptosis, respectively (Fig. 4A–F). CTB proliferation was significantly reduced in explants transfected with p32-specific siRNA sequences (*P < 0.05, *P* < 0.01; Fig. 4G); however, M30-positive CTB were rarely observed in the explants and their numbers were not significantly altered by p32 knockdown (Fig. 4H). LDH release into the culture medium was quantified as an indirect measure of tissue necrosis; LDH release was not significantly changed following p32 knockdown (Fig. 4I).

### p32 knockdown alters glucose extraction and lactate release by term placental explants

**Figure 5:**
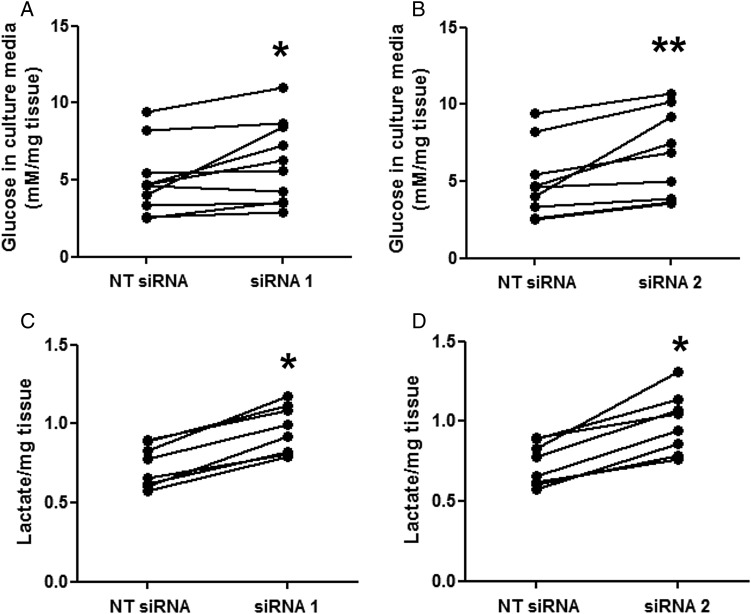
p32 knockdown alters glucose uptake and lactate release. Term placental explants were transfected with a non-targeting siRNA sequence (NT; 50 nM) or two different siRNA sequences specific for p32 (siRNA 1, siRNA 2; 50 nM). After 72 h, (**A** and **B**) glucose and (**C** and **D**) lactate concentrations were measured in the culture medium and normalized to the wet weight of each explant. Lactate concentration is given in arbitrary units. (A and B): Median, *n* = 10; *P < 0.05, **P < 0.01, Wilcoxon matched-pairs test. (C and D): Median, *n* = 8; *P < 0.05, Wilcoxon matched-pairs test.

As p32 regulates the balance between glycolysis and oxidative phosphorylation in tumour cells (Fogal *et al.*, 2010), the concentration of glucose and lactate in explant culture medium was quantified following p32 knockdown. Explants transfected with p32-specific siRNA sequences extracted significantly less glucose from the culture medium (Fig. 5A and B; *P < 0.05, **P < 0.01) but released significantly more lactate (Fig. 5C and D; *P < 0.05) per gram of placental tissue.

### p32 knockdown alters markers of mitochondrial function in term placental explants

**Figure 6:**
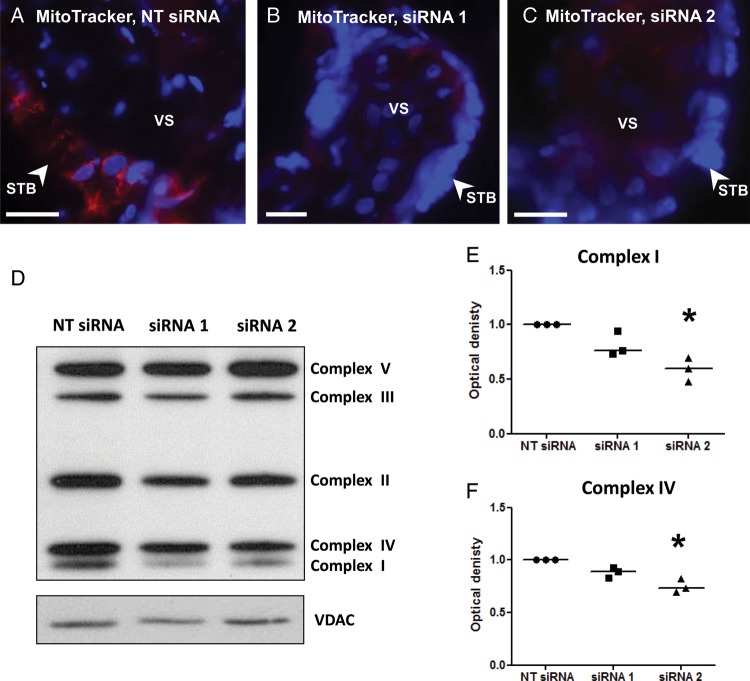
p32 knockdown alters markers of mitochondrial mitochondrial function. (**A**–**C**) Term placental explants were transfected with a non-targeting siRNA sequence (NT; 50 nM) or siRNA sequence 2 (50 nM) for 72 h, then were incubated with MitoTracker for 24 h. The extent of MitoTracker uptake was assessed by fluorescence microscopy. MitoTracker (red); DAPI (nuclei, blue). Images are representative of three independent experiments. Scale bar represents 25 µm. STB, syncytiotrophoblast; VS, villous stroma. (**D**–**F**) Term placental explants were transfected with a non-targeting (NT) or p32-specific siRNA sequences (50 nM) for 72 h. Mitochondrial respiratory complex expression was analysed by western blotting, quantified by densitometry and normalized to expression of the mitochondrial protein VDAC. Median, *n* = 3; *P < 0.05, Kruskal–Wallis test.

Term placental explants were incubated with a MitoTracker dye following p32 knockdown. MitoTracker Red CMXRos is a cell permeable, mitochondrial membrane potential-sensitive fluorochrome that passively diffuses across the plasma membrane and accumulates in active mitochondria. Numerous distinct areas of punctate fluorescence were observed in explants treated with the NT siRNA sequence (Fig. 6A); however, fluorescence was greatly reduced in explants treated with p32-specific siRNA when images were captured at the same exposure (Fig. 6B and C). Expression of voltage-dependent anion channel (VDAC), a ubiquitously expressed mitochondrial porin protein, was unaffected by p32 knockdown suggesting decreased MitoTracker dye accumulation was due to a decrease in active mitochondria rather than a decrease in the number of mitochondria. Supporting this observation, expression of mitochondrial respiratory complexes I and IV were significantly decreased in explants transfected with the p32-specific siRNA sequences (Fig. 6D–F; *P < 0.05), a phenomenon observed in tumour cells following p32 knockdown (Fogal *et al.*, 2010).

## Discussion

In this study, we demonstrate that p32 is highly expressed in the CTB, STB and EVT of human placental tissue throughout gestation and that siRNA-mediated knockdown of p32 in term placental explants correlated with a reduction in CTB proliferation and impairment of mitochondrial function. The majority of tissues use oxidative phosphorylation to generate ATP under aerobic conditions; however, the placenta also generates ATP via aerobic glycolysis, which is characterized by high rates of conversion of glucose to lactate and increased glucose uptake (Bloxam and Bobinski, 1984; Bax and Bloxam, 1997). Knockdown of p32 in tumour cell lines was associated with enhanced glycolysis and increased glucose uptake and lactate production (Fogal *et al.*, 2010). Similarly, lactate release from placental explants was significantly elevated following p32 knockdown; however, glucose extraction from the culture medium was significantly reduced. Rather than being the result of an increase in glycolysis, these changes may reflect an impairment of mitochondrial function: accumulation of MitoTracker dye in placental explants was reduced after p32 knockdown, a process dependent upon the membrane potential of active mitochondria (Pendergrass *et al.*, 2004). A significant decrease in the expression of the mitochondrial respiratory complexes I and IV was also noted following p32 knockdown, as previously reported in tumour cells (Fogal *et al.*, 2010).

We have also shown that p32 expression was significantly reduced in the placentas from women with FGR. Whilst there may be some differences in villous cellular composition between normal and pathological samples, e.g. altered numbers of trophoblast nuclei, a reduced number of arteries and increased stromal extracellular matrix deposition (Giles *et al.*, 1985; Macara *et al.*, 1996), significant differences were not evident from our immunohistochemical analysis. Hence, this is unlikely to account for the observed reduction in p32 expression, which primarily occurred within the trophoblast bilayer. Similarly, altered p32 expression was not related to the gestational age of the FGR placentas, as gestational age at delivery was not significantly different between patient groups.

To recapitulate the reduced syncytial expression of p32 observed in FGR placentas, we used siRNA to knock down p32 expression in the trophoblast bilayer of term placental explants. As p32 knockdown decreased CTB proliferation by ∼50%, reduced p32 expression in FGR placentas may explain some of the pathological features observed, such as reduced CTB turnover and decreased numbers of CTB nuclei (Macara *et al.*, 1996; Chen *et al.*, 2002; Heazell *et al.*, 2010). Interestingly, proteomic analysis of mitochondria isolated from the placentas of women with pre-eclampsia revealed a 5-fold down-regulation of p32 protein expression compared with healthy pregnant women (Shi *et al.*, 2013). Whether altered p32 expression is a cause or a consequence of placental pathology is currently unknown; however, it appears to be indicative of a significant alteration in trophoblast turnover.

Whilst ours is the first study to ascertain the effects of p32 knockdown in human placental explants, ablation of this gene and the effects on fetal development has been studied in mice. The p32 knockout mouse exhibits severe FGR, which presents at embryonic (E) day 10.5 and leads to midgestational lethality between E11.5 and E12.5 (Yagi *et al.*, 2012). BrdU incorporation experiments showed proliferation arrest in E10.5 embryos; however, placental structure and function were not investigated. As observed in our study, expression of several mitochondrial respiratory complexes was reduced in mouse embryonic fibroblasts isolated from p32 knockout pups. In addition, mitochondrial morphology was altered and mitochondrial function was significantly impaired.

C1q, a ligand of p32, has also been identified as an important regulator of placental development and function (Agostinis *et al.*, 2010). The C1q knockout mouse is characterized by reduced fetal weights, smaller litter sizes, decreased trophoblast invasion and spiral artery remodelling and impaired labyrinth formation. These mice also exhibit a pre-eclampsia-like phenotype, presenting with elevated blood pressure, proteinuria, decreased placental vascular endothelial growth factor (VEGF) expression and increased circulating levels of sFlt-1 (Singh *et al.*, 2011). In the first trimester of human pregnancy, C1q is found throughout the decidual stroma, is synthesized by EVT and facilitates interactions with the decidual extracellular matrix (Agostinis *et al.*, 2010). *In vitro*, C1q promoted trophoblast adhesion and migration via interactions with p32 and integrin α4β1.

Association of p32 with the cell membrane has been shown to promote cell migration and invasion in various cell types, in part via interactions with integrins αvβ3, α5 and β1 (Feng *et al.*, 2002; Prakash *et al.*, 2011; Zhang *et al.*, 2013), suggesting that it may also play an important role in the regulation of EVT invasion. As our immunohistochemical analysis revealed p32 expression in first trimester EVT, this may be an interesting avenue for future investigation. However, our current data support a role for p32 as a key regulator of CTB proliferation and mitochondrial function in the villous placenta and suggest that reduced expression of p32 in placentas from complicated pregnancies may contribute to the underlying pathology.

## Supplementary data

Supplementary data are available at http://molehr.oxfordjournals.org/.

## Authors' roles

M.D. and L.K.H. designed the experiments; P.M., J.A.H., F.B. and S.L. performed the research; P.M., J.A.H., F.B., S.L., M.D. and L.K.H. analysed the data; P.M., J.A.H., M.D. and L.K.H. wrote the manuscript. All authors discussed the results and commented on the manuscript.

## Funding

This work was supported by a BBSRC David Phillips Fellowship (BB/H022627/1 to L.K.H.), a project grant from The Wellcome Trust (094361/Z/10/Z), the Manchester Wellcome Trust Clinical Research Facility and the NIHR Greater Manchester Comprehensive Local Research Network. The Maternal and Fetal Health Research Centre is supported by funding from Tommy's the Baby Charity, an Action Research Endowment Fund, the Manchester NIHR Biomedical Research Centre and the NIHR Greater Manchester Comprehensive Local Research Network. Funding to pay the Open Access publication charges for this article was provided by the BBSRC.

## Supplementary Material

Supplementary Data

## References

[GAU039C1] Agostinis C, Bulla R, Tripodo C, Gismondi A, Stabile H, Bossi F, Guarnotta C, Garlanda C, De Seta F, Spessotto P (2010). An alternative role of C1q in cell migration and tissue remodeling: contribution to trophoblast invasion and placental development. J Immunol.

[GAU039C2] Bax BE, Bloxam DL (1997). Energy metabolism and glycolysis in human placental trophoblast cells during differentiation. Biochim Biophys Acta.

[GAU039C3] Bloxam DL, Bobinski PM (1984). Energy metabolism and glycolysis in the human placenta during ischaemia and in normal labour. Placenta.

[GAU039C4] Chen CP, Bajoria R, Aplin JD (2002). Decreased vascularization and cell proliferation in placentas of intrauterine growth-restricted fetuses with abnormal umbilical artery flow velocity waveforms. Am J Obstet Gynecol.

[GAU039C5] Chen YB, Jiang CT, Zhang GQ, Wang JS, Pang D (2009). Increased expression of hyaluronic acid binding protein 1 is correlated with poor prognosis in patients with breast cancer. J Surg Oncol.

[GAU039C6] Dedio J, Jahnen-Dechent W, Bachmann M, Muller-Esterl W (1998). The multiligand-binding protein gC1qR, putative C1q receptor, is a mitochondrial protein. J Immunol.

[GAU039C7] Desforges M, Sibley CP (2009). Placental nutrient supply and fetal growth. Int J Dev Biol.

[GAU039C8] Desforges M, Parsons L, Westwood M, Sibley CP, Greenwood SL (2013). Taurine transport in human placental trophoblast is important for regulation of cell differentiation and survival. Cell Death Dis.

[GAU039C9] Feng X, Tonnesen MG, Peerschke EI, Ghebrehiwet B (2002). Cooperation of C1q receptors and integrins in C1q-mediated endothelial cell adhesion and spreading. J Immunol.

[GAU039C10] Fogal V, Zhang L, Krajewski S, Ruoslahti E (2008). Mitochondrial/cell-surface protein p32/gC1qR as a molecular target in tumor cells and tumor stroma. Cancer Res.

[GAU039C11] Fogal V, Richardson AD, Karmali PP, Scheffler IE, Smith JW, Ruoslahti E (2010). Mitochondrial p32 protein is a critical regulator of tumor metabolism via maintenance of oxidative phosphorylation. Mol Cell Biol.

[GAU039C12] Forbes K, Desforges M, Garside R, Aplin JD, Westwood M (2009). Methods for siRNA-mediated reduction of mRNA and protein expression in human placental explants, isolated primary cells and cell lines. Placenta.

[GAU039C13] Ghebrehiwet B, Peerschke EI (2004). cC1q-R (calreticulin) and gC1q-R/p33: ubiquitously expressed multi-ligand binding cellular proteins involved in inflammation and infection. Mol Immunol.

[GAU039C14] Ghosh I, Chowdhury AR, Rajeswari MR, Datta K (2004). Differential expression of Hyaluronic Acid Binding Protein 1 (HABP1)/P32/C1QBP during progression of epidermal carcinoma. Mol Cell Biochem.

[GAU039C15] Giles WB, Trudinger BJ, Baird PJ (1985). Fetal umbilical artery flow velocity waveforms and placental resistance: pathological correlation. Br J Obstet Gynaecol.

[GAU039C16] Harris LK, Crocker IP, Baker PN, Aplin JD, Westwood M (2010). IGF2 actions on trophoblast in human placenta are regulated by the insulin-like growth factor 2 receptor, which can function as both a signaling and clearance receptor. Biol Reprod.

[GAU039C17] Heazell AE, Sharp AN, Baker PN, Crocker IP (2010). Intra-uterine growth restriction is associated with increased apoptosis and altered expression of proteins in the p53 pathway in villous trophoblast. Apoptosis.

[GAU039C18] Hu M, Crawford SA, Henstridge DC, Ng IH, Boey EJ, Xu Y, Febbraio MA, Jans DA, Bogoyevitch MA (2013). p32 protein levels are integral to mitochondrial and endoplasmic reticulum morphology, cell metabolism and survival. Biochem J.

[GAU039C19] Hulme CH, Westwood M, Myers JE, Heazell AE (2012). A high-throughput colorimetric-assay for monitoring glucose consumption by cultured trophoblast cells and placental tissue. Placenta.

[GAU039C20] Kim KB, Yi JS, Nguyen N, Lee JH, Kwon YC, Ahn BY, Cho H, Kim YK, Yoo HJ, Lee JS (2011). Cell-surface receptor for complement component C1q (gC1qR) is a key regulator for lamellipodia formation and cancer metastasis. J Biol Chem.

[GAU039C21] Levy R, Smith SD, Yusuf K, Huettner PC, Kraus FT, Sadovsky Y, Nelson DM (2002). Trophoblast apoptosis from pregnancies complicated by fetal growth restriction is associated with enhanced p53 expression. Am J Obstet Gynecol.

[GAU039C22] Macara L, Kingdom JC, Kaufmann P, Kohnen G, Hair J, More IA, Lyall F, Greer IA (1996). Structural analysis of placental terminal villi from growth-restricted pregnancies with abnormal umbilical artery Doppler waveforms. Placenta.

[GAU039C23] Mayhew TM (2001). Villous trophoblast of human placenta: a coherent view of its turnover, repair and contributions to villous development and maturation. Histol Histopathol.

[GAU039C24] Murray AJ (2012). Oxygen delivery and fetal-placental growth: beyond a question of supply and demand?. Placenta.

[GAU039C25] Muta T, Kang D, Kitajima S, Fujiwara T, Hamasaki N (1997). p32 protein, a splicing factor 2-associated protein, is localized in mitochondrial matrix and is functionally important in maintaining oxidative phosphorylation. J Biol Chem.

[GAU039C26] Peerschke EI, Ghebrehiwet B (2007). The contribution of gC1qR/p33 in infection and inflammation. Immunobiology.

[GAU039C27] Pendergrass W, Wolf N, Poot M (2004). Efficacy of MitoTracker Green and CMXrosamine to measure changes in mitochondrial membrane potentials in living cells and tissues. Cytometry A.

[GAU039C28] Pijnenborg R, Vercruysse L, Hanssens M (2006). The uterine spiral arteries in human pregnancy: facts and controversies. Placenta.

[GAU039C29] Prakash M, Kale S, Ghosh I, Kundu GC, Datta K (2011). Hyaluronan-binding protein 1 (HABP1/p32/gC1qR) induces melanoma cell migration and tumor growth by NF-kappa B dependent MMP-2 activation through integrin alpha(v)beta(3) interaction. Cell Signal.

[GAU039C30] Rozanov DV, Ghebrehiwet B, Postnova TI, Eichinger A, Deryugina EI, Strongin AY (2002). The hemopexin-like C-terminal domain of membrane type 1 matrix metalloproteinase regulates proteolysis of a multifunctional protein, gC1qR. J Biol Chem.

[GAU039C31] Sengupta A, Banerjee B, Tyagi RK, Datta K (2005). Golgi localization and dynamics of hyaluronan binding protein 1 (HABP1/p32/C1QBP) during the cell cycle. Cell Res.

[GAU039C32] Shi Z, Long W, Zhao C, Guo X, Shen R, Ding H (2013). Comparative proteomics analysis suggests that placental mitochondria are involved in the development of pre-eclampsia. PLoS One.

[GAU039C33] Singh J, Ahmed A, Girardi G (2011). Role of complement component C1q in the onset of preeclampsia in mice. Hypertension.

[GAU039C34] Yagi M, Uchiumi T, Takazaki S, Okuno B, Nomura M, Yoshida S, Kanki T, Kang D (2012). p32/gC1qR is indispensable for fetal development and mitochondrial translation: importance of its RNA-binding ability. Nucleic Acids Res.

[GAU039C35] Yu L, Zhang Z, Loewenstein PM, Desai K, Tang Q, Mao D, Symington JS, Green M (1995). Molecular cloning and characterization of a cellular protein that interacts with the human immunodeficiency virus type 1 Tat transactivator and encodes a strong transcriptional activation domain. J Virol.

[GAU039C36] Yu H, Liu Q, Xin T, Xing L, Dong G, Jiang Q, Lv Y, Song X, Teng C, Huang D (2013). Elevated expression of hyaluronic acid binding protein 1 (HABP1)/P32/C1QBP is a novel indicator for lymph node and peritoneal metastasis of epithelial ovarian cancer patients. Tumour Biol.

[GAU039C37] Zhang X, Zhang F, Guo L, Wang Y, Zhang P, Wang R, Zhang N, Chen R (2013). Interactome analysis reveals that C1QBP is associated with cancer cell chemotaxis and metastasis. Mol Cell Proteomics.

