# Supplementary material for: A role for the mitochondrial-associated protein p32 in regulation of trophoblast proliferation
Source: Mol Hum Reprod. 2014 May 29;20(8):745–55. doi: 10.1093/molehr/gau039 (PMC4106637; doi:10.1093/molehr/gau039)
Supplement: Supplementary Data [file supp_20_8_745__index.html]

A role for the mitochondrial-associated protein p32 in regulation of trophoblast proliferation — A role for the mitochondrial-associated protein p32 in regulation of trophoblast proliferation — Supplementary Data 

# A role for the mitochondrial-associated protein p32 in regulation of trophoblast proliferation

## Supplementary Data

Supplementary Data

**Files in this Data Supplement:**

- Supplementary Data - Doc file
- Supplementary Figure 1 - pptx file
- Supplementary Figure 2 - pptx file
- Supplementary Figure 3 - pptx file
