# Supplementary material for: A role for the mitochondrial-associated protein p32 in regulation of trophoblast proliferation
Source: Mol Hum Reprod. 2014 May 29;20(8):745–55. doi: 10.1093/molehr/gau039 (PMC4106637; doi:10.1093/molehr/gau039)
Supplement: Supplementary Data [file supp_gau039_gau039supp.doc]

**Supplementary Figure 1: β-actin expression in first trimester and term placental tissue**

β-actin protein expression in first trimester and term placental tissue was analysed by western blotting and quantified by densitometry. Median, n=6.

**Supplementary Figure 2: p32 protein expression in human placenta (expanded images)**

Immunohistochemical analysis of [A-D] first trimester, [E-G] second trimester and [H] term placenta stained with an antibody to p32 (diaminobenzidine (DAB) labelling, brown). Nuclei were counterstained with haematoxylin (blue). Scale bar represents 50µm. Images are representative of n=12 samples.

**Supplementary Figure 3: p32 mRNA expression in human placenta**

p32 mRNA expression in first trimester and term placenta was assessed by quantitative PCR and normalised to expression of the housekeeping gene tyrosine 3-monooxygenase/tryptophan 5-monooxygenase activation protein, zeta polypeptide (YWHAZ; median, n=6; **P<0.01, Mann Whitney U test).
