# Supplementary figures and images for: A role for the mitochondrial-associated protein p32 in regulation of trophoblast proliferation
Source: Mol Hum Reprod. 2014 May 29;20(8):745–55. doi: 10.1093/molehr/gau039 (PMC4106637; doi:10.1093/molehr/gau039)

## Slide 1
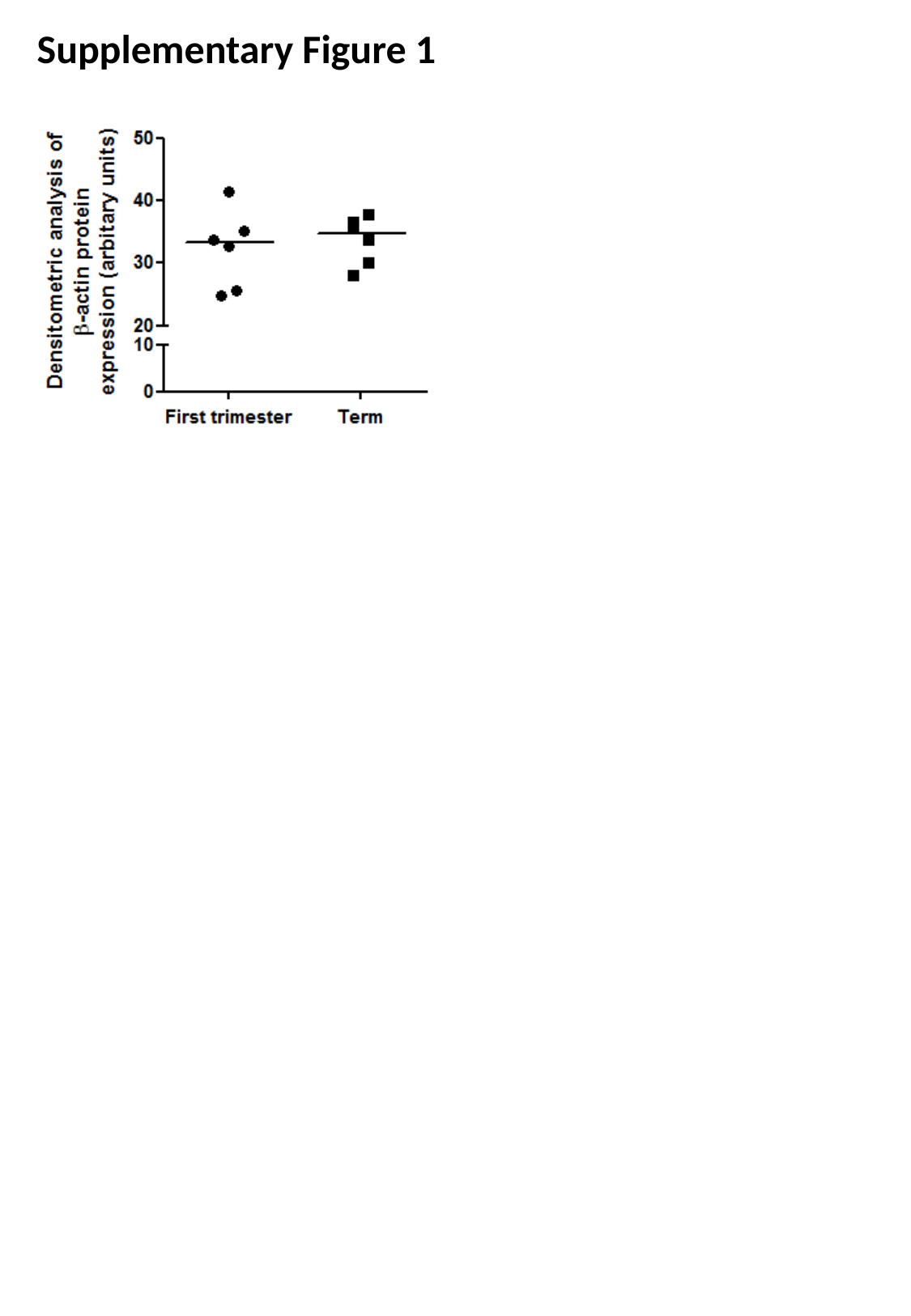

Supplementary Figure 1

Supplement: Supplementary Data [file supp_gau039_gau039supp_fig1.pptx]

## Slide 1
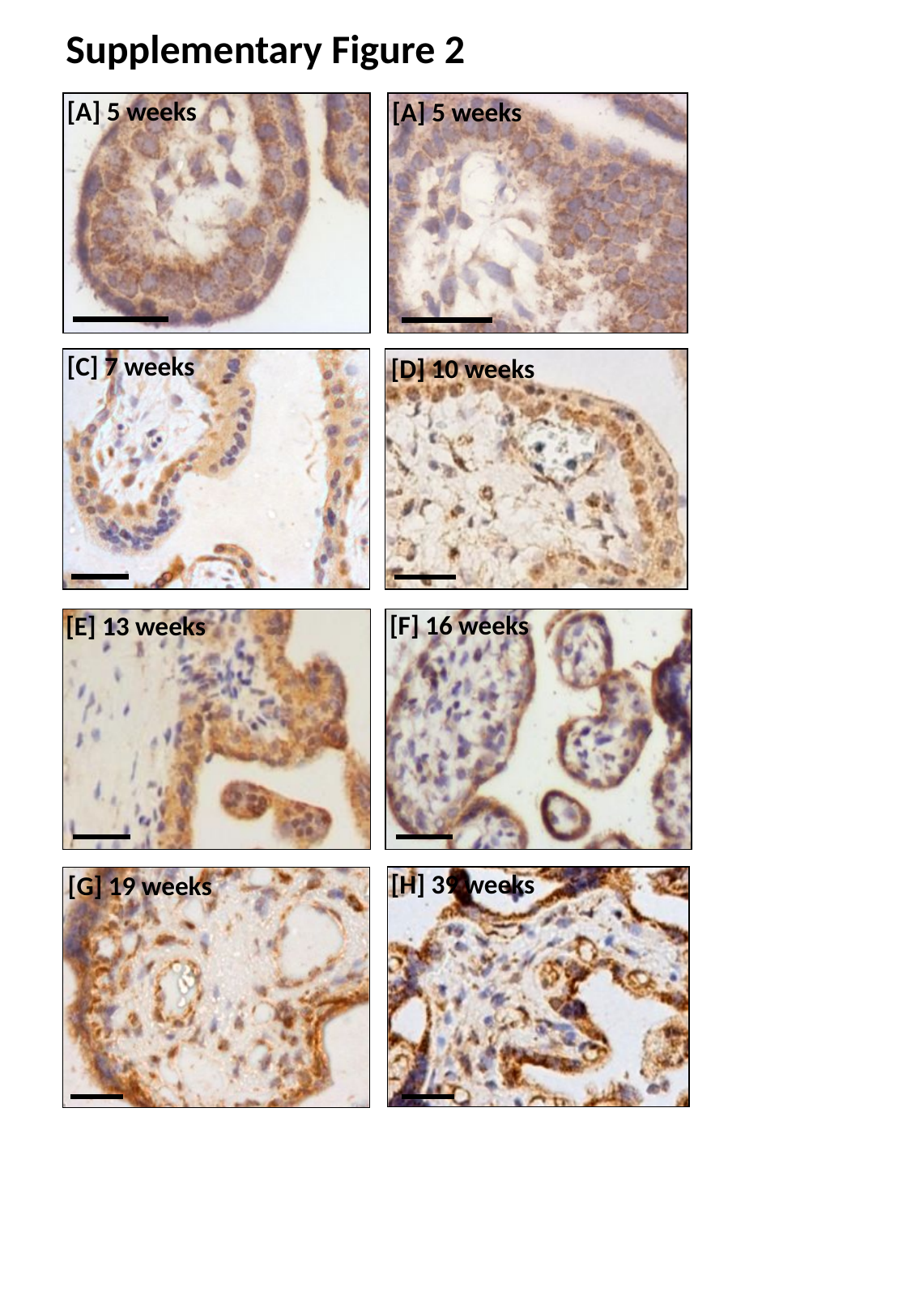

Supplementary Figure 2
[A] 5 weeks
[A] 5 weeks
[C] 7 weeks
[D] 10 weeks
[F] 16 weeks
[E] 13 weeks
[H] 39 weeks
[G] 19 weeks

Supplement: Supplementary Data [file supp_gau039_gau039supp_fig2.pptx]

## Slide 1
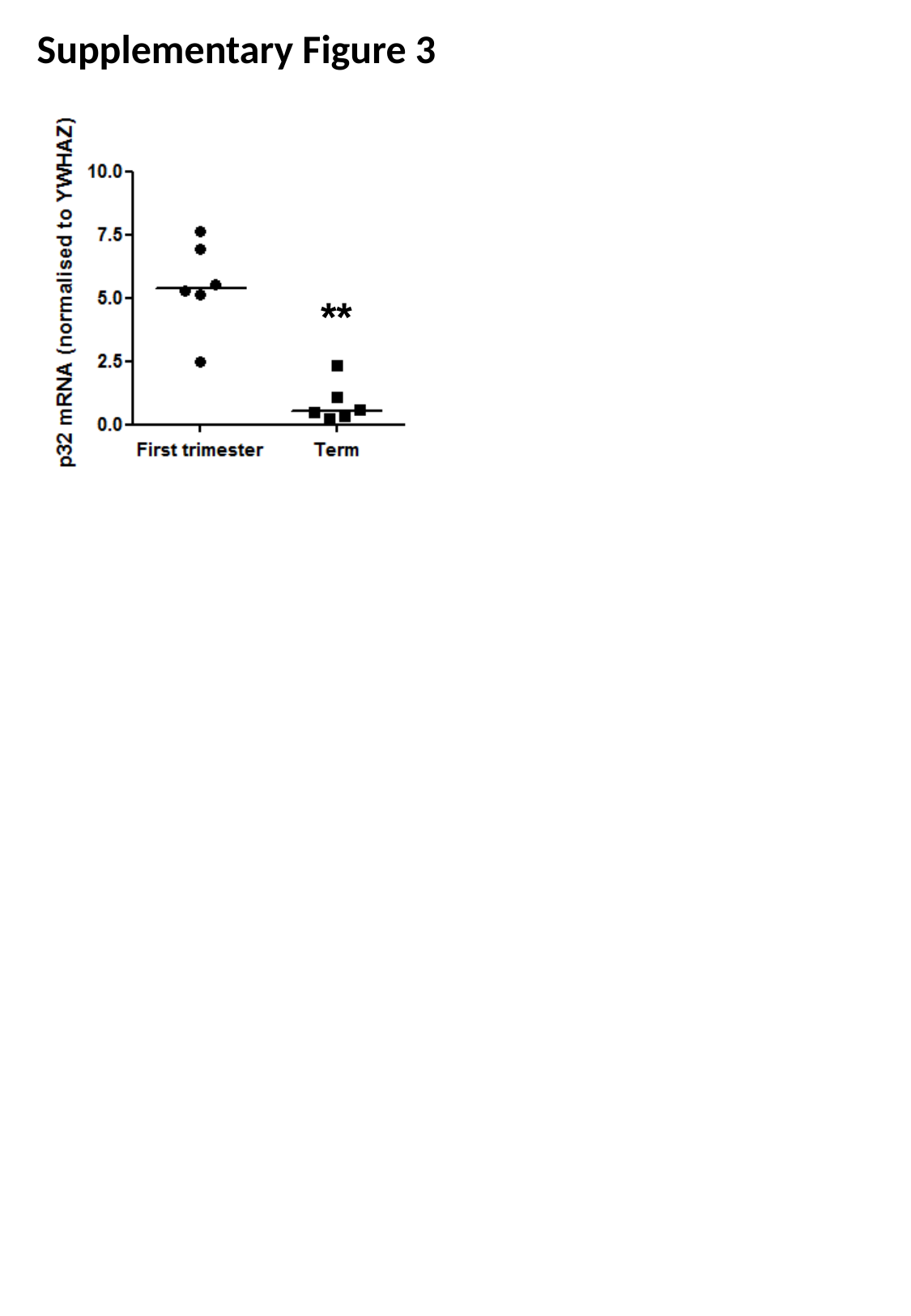

Supplementary Figure 3
**

Supplement: Supplementary Data [file supp_gau039_gau039supp_fig3.pptx]
